# Immunotherapy- (Blinatumomab-) Related Lineage Switch of *KMT2A/AFF1* Rearranged B-Lymphoblastic Leukemia into Acute Myeloid Leukemia/Myeloid Sarcoma and Subsequently into B/Myeloid Mixed Phenotype Acute Leukemia

**DOI:** 10.1155/2019/7394619

**Published:** 2019-12-07

**Authors:** Rui R. He, Zacharia Nayer, Matthew Hogan, Raymund S. Cuevo, Kimberly Woodward, David Heyer, Christine A. Curtis, Jess F. Peterson

**Affiliations:** ^1^Department of Pathology, Inova Fairfax Hospital, Falls Church, VA, USA; ^2^School of Medicine, George Washington University, Washington, DC, USA; ^3^School of Medicine, Virginia Commonwealth University, Richmond, VA, USA; ^4^Inova Schar Cancer Institute, Inova Fairfax Hospital, Falls Church, VA, USA; ^5^Department of Cytogenetics, Quest Diagnostics Nichols Institute, Chantilly, VA, USA; ^6^Division of Laboratory Genetics and Genomics, Department of Laboratory Medicine and Pathology, Mayo Clinic, Rochester, MN, USA

## Abstract

The presence of *KMT2A/AFF1* rearrangement in B-lymphoblastic leukemia (B-ALL) is an independent poor prognostic factor and has been associated with higher rate of treatment failure and higher risk of linage switch under therapy. Blinatumomab has shown promising therapeutic results in refractory or relapsed B-ALL; however, it has potential risk of inducing lineage switch, especially in *KMT2A/AFF1* rearranged B-ALL into acute myeloid leukemia and/or myeloid sarcoma. We report a 40-year-old female with *KMT2A/AFF1*-rearranged B-ALL that was refractory to conventional chemotherapy. Following administration of blinatumomab, she developed a breast mass proven to be myeloid sarcoma, in addition to bone marrow involvement by AML. Approximately six weeks after cessation of blinatumomab, a repeat bone marrow examination revealed B/myeloid MPAL.

## 1. Introduction

Immunotherapy targeted at CD19, either antibody-based (blinatumomab) or T-cell mediated (CAR T cells), represents a promising treatment strategy for patients with refractory B-lymphoblastic leukemia (B-ALL). Early phase clinical trials have shown high rates of complete remission in refractory pediatric B-ALL patients after CD19 CAR-T-cell or blinatumomab therapy [1, 2]. However, a rare event, lineage switch from B-ALL to acute myeloid leukemia (AML) can occur following CD19 targeted therapy, most commonly in *KMT2A*-rearranged B-ALL [3–6]. The *KMT2A* gene is a critical target of chromosomal rearrangements observed in ALL, AML, mixed phenotype acute leukemia (MPAL), and therapy-related myeloid neoplasms [7]. The presence of *KMT2A* rearrangement, especially in B-ALL, has long been associated with a higher risk of lineage switch under therapy and is an independent dismal prognostic factor [8]. However, the exact mechanism and management of linage switch events are unclear. Herein, we report a 40-year-old female with *KMT2A/AFF1*-rearranged B-ALL refractory to conventional chemotherapy. Following administration of blinatumomab, she developed a breast mass proven to be myeloid sarcoma, in addition to bone marrow involvement by AML. Approximately six weeks after cessation of blinatumomab, a repeat bone marrow examination revealed B/myeloid MPAL.

## 2. Case Description

**Figure 1:**
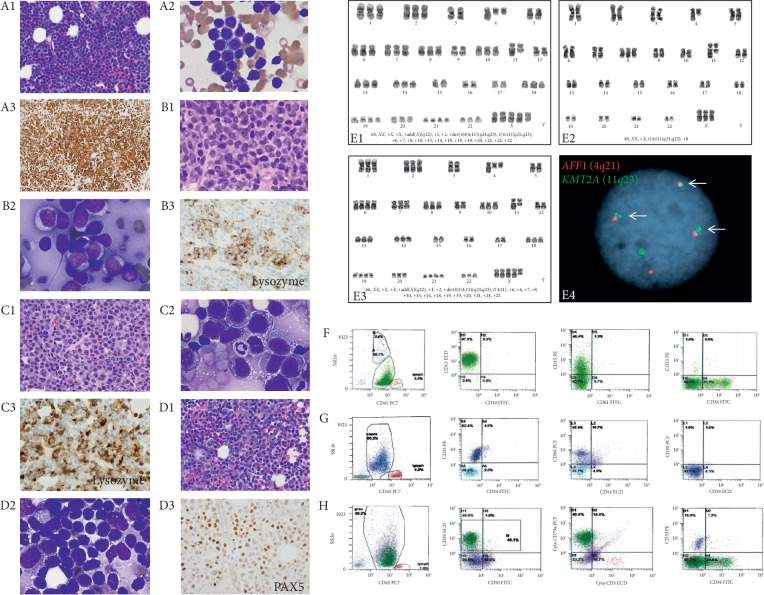
Morphologic, immunohistochemical, flow cytometric, and cytogenetic characteristics of the patient's leukemia. A1–A3 represent bone marrow evaluation at initial diagnosis. Bone marrow biopsy (A1, H&E, 400x) and aspirate (A2, Wright stain 100x, oil) showing numerous small-sized B-lymphoblasts which are strongly positive for PAX5 (A3). B1–B3 represent biopsy of the breast mass (B1, H&E, 400x) and touch imprint (B2, Wright stain, 100x, oil) showing numerous large-sized blasts with monocytic differentiation, which are patchy positive for lysozyme (B3). C1–C3 represent bone marrow biopsy (C1, H&E, 400x) and aspirate (C2, Wright stain, 100x, oil) showing sheets of myeloblasts which are patchy positive for lysozyme (C3). D1–D3 represent bone marrow evaluation six weeks after cessation of blinatumomab. Core biopsy (D1, H&E, 400x) and aspirate (D2, Wright stain, 100x, Oil) show a dimorphic population of blasts: small-sized B-lymphoblasts which are positive for PAX5 (D3) and large-sized myeloblasts which are positive for lysozyme (data not shown here). E1 represents the karyogram of bone marrow specimen at myeloblastic transformation. E2–E4 represent karyograms and *AFF1/KMT2A* fusion of bone marrow specimen with B/myeloid mixed phenotype acute leukemia. F–H represent flow cytometric features of the leukemic blasts. F represents flow cytometry performed on the bone marrow aspirate at the initial diagnosis showing a large population of B-lymphoblasts (green) in dim CD45 region expressing CD19, CD34 (partial), and CD15 (dim), G represents flow cytometry of the bone marrow aspirate while administration of blinatumomab showing a population of myeloblasts (blue) expressing CD33 and CD64 (dim) and was negative for CD19 and CD34. H represents flow cytometry of the bone marrow aspirate six weeks after cessation of blinatumomab showing two populations: B-lymphoblasts (green) expressing CD19, CD34 (dim), and cytoCD79a, and myeloblasts (blue) expressing CD33, CD64 (dim), and MPO (data not shown here).

A 40-year-old female without a past medical history presented with two weeks of easy bruising, fatigue, and muscle aches. A complete blood count revealed leukocytosis (white blood cell count, 71.8 × 10^3^/*μ*L; reference, 3.4–9.6 × 10^3^/*μ*L), anemia (hemoglobin, 12.6 g/dL; reference, 13.2–16.6 g/dL), and thrombocytopenia (platelet count, 77 × 10^3^/*μ*L; reference, 135–317 × 10^3^/*μ*L). Peripheral blood smear revealed numerous small-to-intermediate-sized blasts with high nuclear-to-cytoplasmic (N : C) ratio, fine chromatin, and prominent nucleoli. Flow cytometry performed on peripheral blood sample revealed a large population of blasts in the dim CD45 region expressing CD19 (bright), CD34 (dim), and CD15 (dim) and was negative for CD10. A subset of blasts appeared to be positive for myeloperoxidase (MPO). Bone marrow evaluation revealed a hypercellular bone marrow (90%) composed of numerous small-to-intermediate-sized blasts with similar morphology as the blasts are identified in peripheral blood smear (Figure 1, A1 and A2). Flow cytometry of bone marrow aspirate revealed a large population of blasts immunophenotypically identical to the blasts detected in peripheral blood (Figure 1, F). Since it was questionable for MPO positivity in a subset of blasts, immunohistochemical analysis was performed on the bone marrow biopsy specimen. The blasts were strongly positive for PAX5 (Figure 1, A3), CD19, and CD79a; focally positive for CD34; but were completely negative for MPO (data not shown). Taken together, these findings are consistent with a diagnosis of B-ALL. Chromosomal analysis revealed a complex karyotype including *t*(4; 11)(*q*21; *q*23), while fluorescence *in situ* hybridization (FISH) confirmed *KMT2A* rearrangement (Table 1). The patient received induction chemotherapy with cyclophosphamide, daunorubicin, vincristine, and dexamethasone (HyperCVAD cycle 1A). A month after her blood cell count recovery, she was found to have circulating blasts and was treated with HyperCVAD cycle 1B (with high dose cytarabine and methotrexate) but had resistant disease on day 21 of therapy. She was started on salvage blinatumomab the next day and treated per protocol. The patient developed cytokine release syndrome and was treated transiently. A month into blinatumomab therapy, the patient subsequently developed a painful right breast mass. The biopsy from the mass showed sheets of large-sized blastic cells (Figure 1, B1 andB2) which were positive for lysozyme (B3) but negative for CD19, PAX5, and other B-cell markers (data not shown) by immunohistochemistry, consistent with a diagnosis of myeloid sarcoma. A bone marrow evaluation revealed a hypercellular marrow (90%) composed of sheets of blasts with monocytic features (Figure 1, C1 andC2). Flow cytometry from the bone marrow aspirate detected a population of blasts expressing CD33 and CD64 (dim), but was negative for CD19 and CD34 (Figure 1, G). The blasts were also positive for CD13 and myeloperoxidase and negative for cytoCD79a (data not shown). Immunohistochemical study performed on bone marrow biopsy showed the blasts were positive for lysozyme (Figure 1, C3). Taken together, a diagnosis of AML with monocytic differentiation was rendered. Conventional chromosome analysis from the bone marrow aspirate revealed *t*(4; 11)(*q*21; *q*23) and additional chromosomal abnormalities (Figure 1, E1; Table 1). Six weeks after discontinuation of blinatumomab, a repeat bone marrow biopsy and aspirate demonstrated a hypercellular marrow (80%) with a dimorphic population of blasts composed of mixed small-and large-sized blasts (Figure 1, D1-D2). Flow cytometry and immunohistochemical studies confirmed the presence of two populations of blasts: (1) B-lymphoblasts phenotypically identical to those in the patient's initial bone marrow specimen expressing CD19, CD34 (dim), and cytoCD79a and (2) myeloblasts phenotypically similar to those in her second bone marrow specimen expressing CD33 and CD64 (Figure 1, H). The myeloblasts were also positive for myeloperoxidase (data not shown). These findings indicated a diagnosis of B/myeloid MPAL. Chromosome and FISH studies confirmed the presence of *KMT2A/AFF1* fusion with additional chromosomal abnormalities (Figure 1, E2–E4; Table 1). The patient's leukemia did not respond to mitoxantrone, etoposide, cytarabine (MEC), and fludarabine, cytarabine, and idarubicin with growth factor (FLAG-ida), and she passed away on day 12 of her last regimen.

## 3. Discussion

We present a case of a 40-year-old female with an initial diagnosis of *KMT2A/AFF1* rearranged B-ALL that subsequently switched to more aggressive types of leukemic events and with extramedullary involvement. The unique features of this case include several clonally related, but phenotypically distinct leukemic events (B-ALL, AML, myeloid sarcoma of the breast, and MPAL) that occurred within a six-month period. The transformed AML/myeloid sarcoma and MPAL were associated with administration and cessation of blinatumomab, respectively, and demonstrated additional cytogenetic abnormalities in addition to *KMT2A/AFF1* fusion. This case provides evidence that two key factors appear to be involved in this lineage switching event: *KMT2A* rearrangement and blinatumomab therapy. *KMT2A*-rearranged acute leukemia represents a heterogeneous group of disease overlapping lymphoid and myeloid with more than 100 different fusion partners identified to date [6]. The presence of *KMT2A* rearrangement has long been associated with a higher risk of lineage switch under chemotherapy and subsequent failure to treatment even before the emergence of immunotherapies [8, 9]. As a monoclonal antibody with bispecificity for both CD19 on B cells and CD3 on cytotoxic T cells, blinatumomab has shown promising therapeutic results in treating refractory or relapsed B-ALL; however, the risk of inducing lineage switch especially in *KMT2A/AFF1* rearranged B-ALL should not be underestimated.

While the exact mechanism of linage switch remains unclear, several possible mechanisms have been proposed [9–14]. Studies have suggested that inherent lineage plasticity of early progenitor cells and immunotherapeutic pressure-induced lineage reprogramming play important roles [3, 9, 10]. An experimental study demonstrated that cellular microenvironment affects cell fate decisions and lineage interconversions [12]. Other studies hypothesized that immunotherapy-induced cytokine release (notably interleukin 6) may promote myeloid differentiation of a lymphoid clone [1, 3, 13]. Additional studies have postulated that genetic evolutions of leukemic blasts under targeted therapy may contribute to lineage switch [10, 14]. The findings in this case suggest that high biphenotypic potential of *KMT2A/AFF1* rearranged B-ALL blasts, blinatumomab-induced blastic cell reprogramming, clonal evolution, and cytokine release might have played important roles in this lineage switching event.

In conclusion, the lineage switch events indicate that cautious application of immunotherapy in *KMT2A/AFF1*-rearranged B-ALL should be advocated in clinical settings. Targeting multiple antigens on leukemia-initiating cells may be a better strategy to reduce the likelihood of lineage switching.

## Figures and Tables

**Table 1:** Genetic results obtained at the time of B-ALL diagnosis, transformation to AML following initiation of blinatumomab therapy, and subsequent posttransformation to MPAL upon discontinuation of blinatumomab.

Genetic testing	Diagnosis: B-ALL (10/26/2018)	Transformation: AML (02/25/2019)	Transformation: MPAL (04/18/2019)
Conventional chromosome analysis	48, XX, +X, *t*(4; 11)(*q*21; *q*23), +8[20]/49, idem, +X, *i*(X) (*p*10)*x*2, −8[2]/46, XX[1]	63∼67, XX, +X, +X, +add(X)(*q*22)*x*1∼2, +1, +2, *t*(4; 11)(*q*21; *q*23), +der(4)*t*(4; 11), +6, +7, +8, +10, +13, +14, +18, +19, +19, +20, +21, +21, +22[*cp*10]/46, XX[10]	48, XX, +X, *t*(4; 11)(*q*21; *q*23), +8[18]/64∼66, idem, +X, +add(X)(*q*22), +1, +2, +der(4) *t*(4; 11), +6, +6, +7, +10, +13, +14, +18, +19, +19, +20, +21, +21, +22[*cp*2]

FISH	*KMT2A* rearrangement (93% of 100 interphase nuclei)	Not performed	*AFF1/KMT2A* fusion (92% of 500 interphase nuclei)

B-ALL, B-acute lymphoblastic leukemia; AML, acute myeloid leukemia; MPAL, mixed phenotype acute leukemia; FISH, fluorescence *in situ* hybridization.
